# The Hidden Culprit Unraveled by Dermoscopy: A Rare Case of Pediatric Facial Demodicosis

**DOI:** 10.7759/cureus.103581

**Published:** 2026-02-14

**Authors:** Chaimae Bouhamdi, Hanane Baybay, Meryem Soughi, Sara Elloudi, Fatima Zahra Mernissi

**Affiliations:** 1 Department of Dermatology, Hassan II University Hospital, Fez, MAR

**Keywords:** demodex blepharitis, diagnostic dermoscopy, papulopustular facial eruption, pediatric demodicosis, steroid-exacerbated dermatoses

## Abstract

We report a rare case of pediatric facial and ocular demodicosis in an immunocompetent seven-year-old with chronic centrofacial papulopustular lesions and recurrent blepharitis misdiagnosed over several years. Dermoscopic examination revealed a high-density Demodex infestation on facial skin and eyelashes, leading to a definitive diagnosis. A combined treatment approach using topical and systemic metronidazole, along with targeted ocular therapy, achieved complete remission. This case highlights the diagnostic power of dermoscopy and underscores the importance of considering demodicosis in children with refractory facial and ocular involvement.

## Introduction

Demodicosis is a chronic inflammatory dermatosis of the pilosebaceous unit resulting from the pathogenic proliferation of Demodex mites, which are commensal ectoparasites of human skin [1,2]. While the condition is well characterized in adults, particularly in the context of rosacea or immunosuppression, pediatric demodicosis remains a rare and frequently overlooked entity [3,4]. Its clinical manifestations, including erythema, follicular scaling, papulopustular lesions, and pruritus, often mimic common pediatric dermatoses, such as acne vulgaris, eczema, or seborrheic dermatitis [5,6]. Ocular involvement further complicates the clinical picture, presenting as chronic blepharitis or conjunctivitis often resistant to standard treatment [7,8]. Misdiagnosis contributes to prolonged morbidity and unnecessary therapeutic trials [3,4].

Standard diagnostic techniques for demodicosis include standardized skin surface biopsy, superficial skin scraping, lash epilation, and light microscopic examination, which allow quantitative assessment of mite density [1,2]. However, these methods require sampling procedures that may be uncomfortable or poorly tolerated in pediatric patients. Dermoscopy, on the other hand, provides a rapid, non-invasive bedside tool that enables direct visualization of characteristic features, such as gelatinous cylindrical Demodex tails, follicular plugging, and perifollicular erythema without the need for invasive sampling [1,2]. Its immediate applicability and procedural tolerability make it particularly advantageous in children, where cooperation and minimization of discomfort are essential considerations.

We present a rare case of pediatric facial and ocular demodicosis in an immunocompetent child, diagnosed using dermoscopy after years of inconclusive management. This case highlights the diagnostic value of dermoscopy and underscores the need for increased recognition of this condition in children.

## Case presentation

A seven-year-old child presented with a history of chronic, relapsing-remitting centrofacial inflammatory papules and pustules since birth, without episodes of flushing. The mother noted microcysts and comedones during the first year of life. The child also experienced intermittent eyelid itching and burning, along with recurrent blepharitis, though without signs of ocular dryness.

The patient was assessed by several pediatricians who suspected food or aeroallergen sensitivities. An allergologic workup (Mediwiss Panel 30, Germany) revealed borderline sensitization to egg yolk (F75), soy (F49), and date palm pollen (T14), but no clinically relevant allergen reactivity. Total serum IgE was within normal limits. Despite this, the child remained on restrictive diets. Treatments included moderate-potency topical corticosteroids (prednisolone aceponate) and topical oxytetracycline hydrochloride, both applied intermittently for extended periods.

Multiple ophthalmologists also evaluated the patient, prescribing ophthalmic fusidic acid with partial and transient improvement. A private dermatologist discontinued topical agents and considered differential diagnoses, including infantile acne--rare at this age without retentional lesions--rosacea without flushing, and sarcoidosis. The patient was referred to our tertiary hospital for expert evaluation before considering hormonal testing.

**Figure 1:**
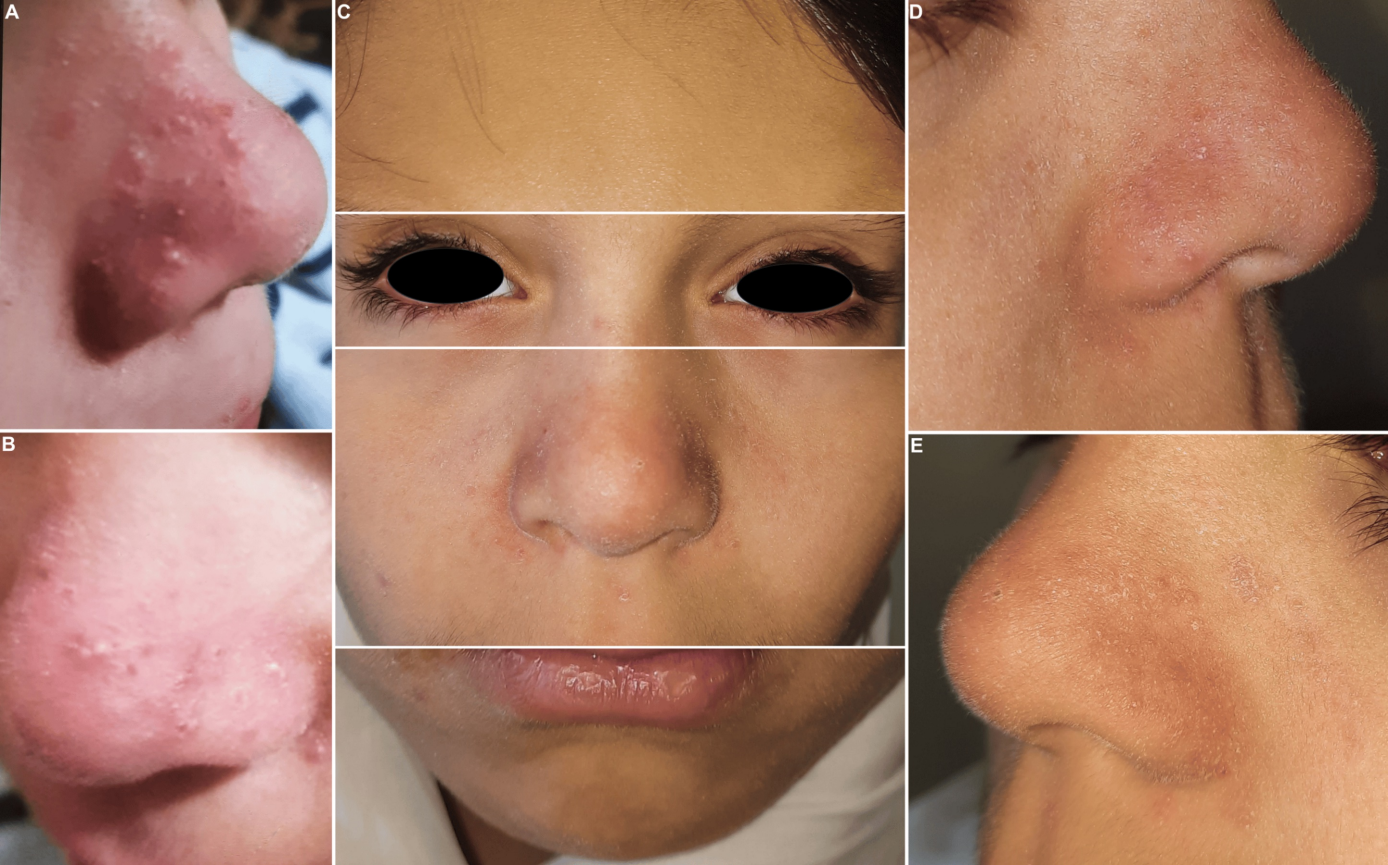
Clinical features. (A) and (B) Parental photographs showing centrofacial papulopustules. (C)-(E) Initial examination revealing xerosis, centrofacial erythema, and papulopustules, with absence of comedones. Eyelid inspection showed signs of chronic blepharitis.

Dermatological examination revealed xerotic skin, centrofacial erythema, and papulopustules on the nasal bridge, perinasal folds, and cheeks. No comedones or lesions elsewhere were observed. Eyelid inspection showed signs of chronic blepharitis (Figure 1).

**Figure 2:**
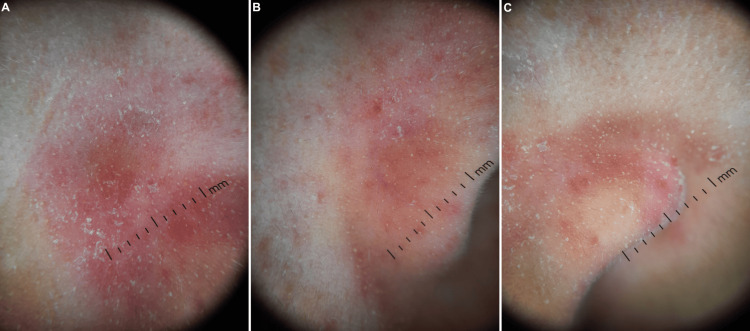
Dermoscopic features. (A) Cheek, (B) right nasal ala, and (C) left nasal ala. (A)-(C) Scaling. High density of Demodex mites presenting as gelatinous cylindrical tails protruding from follicular openings. Follicular pointing with follicular plugs encircled by erythematous halos. Dilated blood vessels. Erythema and pseudo-lupoid pattern.

**Figure 3:**
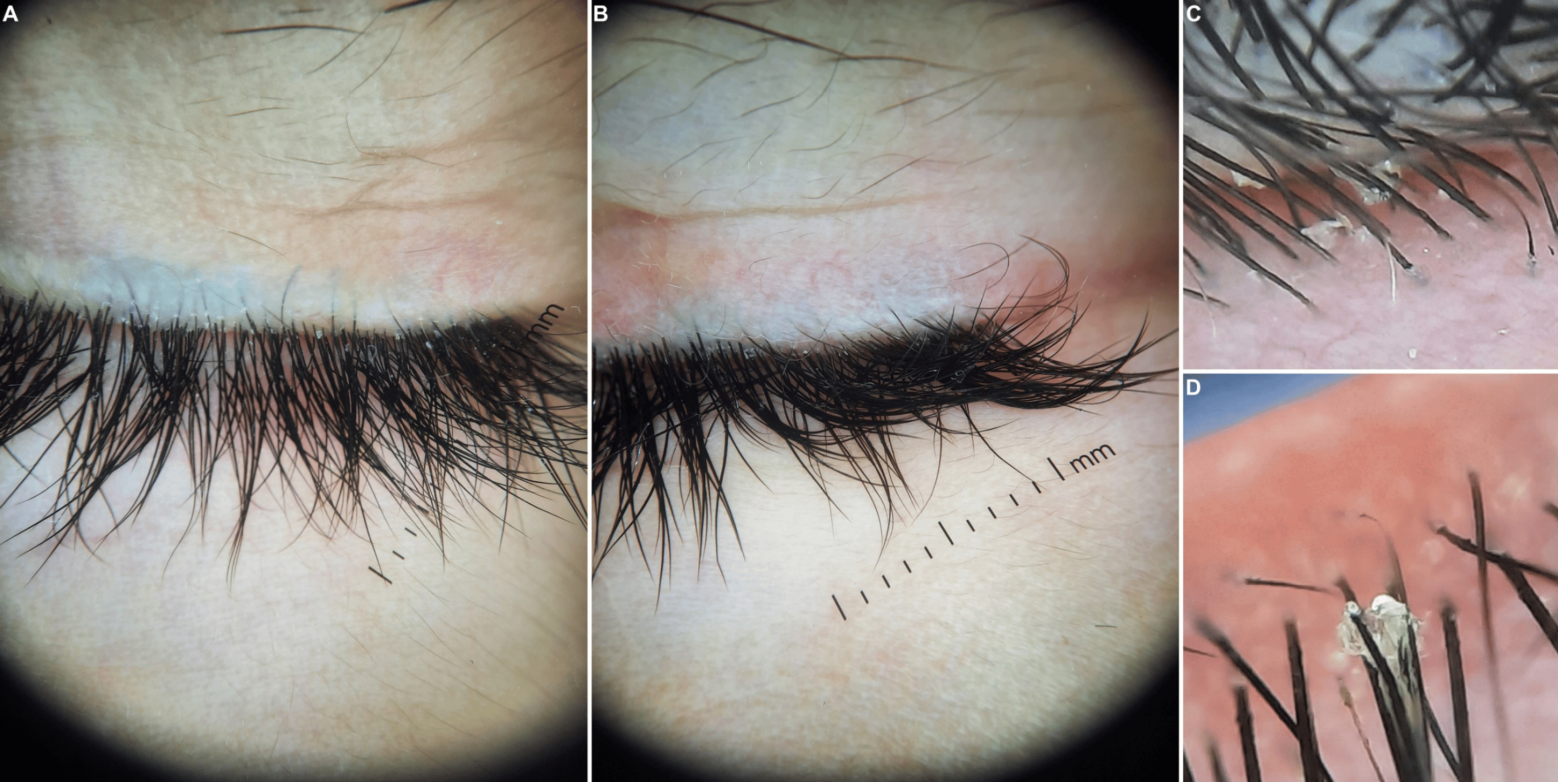
Dermoscopy of the eyelid margins. (A) and (B) Multiple cylindrical sleeves at the base of the lashes. (C) and (D) Isolated Demodex mites anchored perpendicularly to a lash with a fully visible translucent body.

Dermoscopy was performed using a DermLite DL5 (3Gen Inc., USA) at ×10 magnification under polarized light. It identified Demodex mites as gelatinous cylindrical tails protruding from follicular openings, with a density exceeding the commonly accepted pathogenic threshold of >5 mites/cm² described in standardized skin surface biopsy studies [1,9]. Although formal quantitative sampling was not performed, the high density of visible Demodex mites on dermoscopy was considered consistent with clinically significant infestation. Other dermoscopic features included follicular plugging, widened ostia with gray-brown plugs encircled by erythematous halos, follicular pointing, and dilated blood vessels around the nostrils. Some zones had a pseudo-lupoid appearance (Figure 2). Dermoscopy of the eyelashes revealed multiple cylindrical sleeves at the base of the lashes, consistent with ocular demodicosis (Figure 3).

A diagnosis of cutaneous and ocular demodicosis in an immunocompetent child was established. Ophthalmologic treatment included azithromycin 1.5% eye drops (twice daily, in three 15-day cycles), trehalose-based artificial tears (four times daily), and daily warm compresses with lid massage. Cutaneous management included a compounded topical metronidazole applied once daily for three months. Oral metronidazole syrup (125 mg/5 mL) was administered at one teaspoon (5 mL) three times daily for 15 days per month over three months.

**Figure 4:**
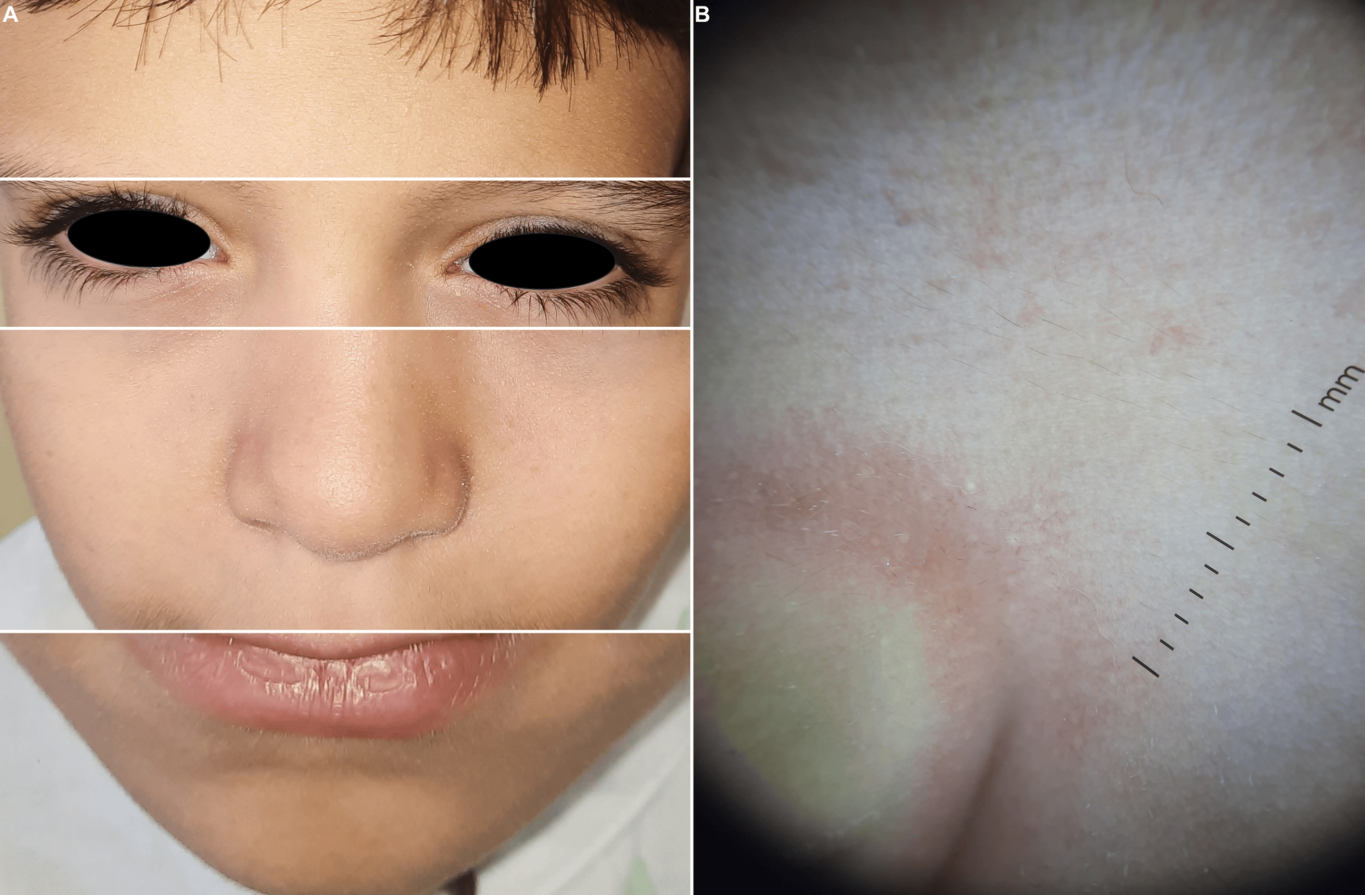
Three-month follow-up. (A) Complete clinical resolution. (B) Absence of Demodex tails in dermoscopy.

At the three-month follow-up, the patient and parents reported complete resolution of facial flares and disappearance of eyelid itching and burning. Examination showed no erythema or active lesions, and eyelid margins were clinically quiet. Repeat dermoscopy confirmed the absence of Demodex tails on both the face and eyelashes (Figure 4).

Maintenance therapy included topical metronidazole for one month, followed by adapalene 0.1% gel every other evening for one month, then nightly for three months. Eyelid care included daily palpebral emollient, a non-soap body cleanser, and a lipid-replenishing dermocosmetic cleanser for the face, applied one hour after adapalene.

In summary, the patient developed persistent centrofacial inflammatory lesions beginning in early infancy, accompanied over time by recurrent blepharitis. Over several years, multiple pediatric and ophthalmologic evaluations led to allergologic testing and repeated empirical treatments, including topical corticosteroids, topical antibiotics, dietary restrictions, and ophthalmic fusidic acid, with only transient or partial improvement. At age seven, referral to our tertiary center enabled dermoscopic assessment, which demonstrated high-density Demodex infestation involving both facial skin and eyelashes. Targeted cutaneous and ocular therapy was subsequently initiated, resulting in complete clinical and dermoscopic resolution within three months.

Written informed consent was obtained from the patient’s parent/legal guardian for publication of clinical details and any accompanying images. A written and signed consent statement was provided to the journal.

## Discussion

Demodicosis, though widely documented in adults, is underrecognized in pediatric populations, especially among immunocompetent children. While Demodex mites are ubiquitous commensals of human skin, their pathogenic overproliferation can provoke a spectrum of inflammatory dermatoses. In children, facial demodicosis often mimics acne, eczema, seborrheic dermatitis, or rosacea, while ocular involvement may present as chronic blepharitis or conjunctivitis unresponsive to standard treatments. These overlapping clinical patterns contribute to diagnostic delays and inappropriate therapies. In the largest pediatric case series available, Douglas and Zaenglein reported only five cases of facial demodicosis, underscoring its rarity and diagnostic complexity [4]. Our case further emphasizes the necessity of including demodicosis in the differential diagnosis of chronic centrofacial eruptions and relapsing blepharitis in children.

The current case illustrates the typical clinical course of pediatric demodicosis: chronicity, partial responses to empirical treatments, and misdiagnoses across multiple specialties. The absence of flushing or comedonal lesions led to inconsistent hypotheses, including allergic dermatitis, infantile acne, and even lupus. Similar diagnostic uncertainty was reported in the series by Izri et al. and Guerrero-González et al., in which affected children underwent extensive workups prior to diagnosis via standardized skin surface biopsy or dermoscopy [3,9]. Importantly, our patient had been subjected to prolonged topical corticosteroids and antibiotics--a therapeutic pattern mirrored in Guzman et al.’s report, where steroid misuse exacerbated Demodex-induced pustulosis [10].

Facial demodicosis in children often presents with erythema, papulopustules, and follicular scaling predominantly in sebaceous-rich areas. In our patient, the presence of follicular accentuation and partial central clearing raised suspicion for a lupoid pattern, echoing features described in Uzuncakmak et al.’s pediatric report of demodicosis mimicking cutaneous lupus [5]. These pseudo-lupoid patterns, though rare, reinforce the importance of clinicopathological correlation and non-invasive visualization tools before advancing to immunologic or histopathologic investigations.

Dermoscopic assessment played a pivotal role in the diagnosis. Visualization of pathognomonic cylindrical Demodex tails emerging from follicular openings, along with dilated follicular ostia containing brownish plugs and perifollicular erythema, confirmed the diagnosis with high specificity [2,11]. The diagnostic threshold for cutaneous demodicosis remains a mite density exceeding 5 per cm² [1], which was observed in our case. In ocular involvement, the identification of cylindrical dandruff at the lash base, as seen in our patient, is highly suggestive of Demodex blepharitis [8,12]. Although mite count does not always correlate with symptom severity, the presence of collarettes or cylindrical sleeves is considered diagnostic. These structures represent retained keratinized debris, unexcreted mite waste, and dead organisms encasing the lash base [8].

The ocular manifestations of demodicosis in children remain poorly recognized. In Liang et al.'s seminal study, Demodex was identified as a potential etiology in pediatric blepharoconjunctivitis, with chronic eyelid inflammation preceding diagnosis by several years [8]. Patel et al. reported similar findings in their case series of young patients, highlighting recurrent blepharokeratoconjunctivitis as a typical but overlooked presentation [7]. Our case supports these observations and demonstrates that a single dermoscopic evaluation of the lash margin can provide the diagnostic breakthrough missed by multiple prior ophthalmologic assessments.

Compared with previously reported pediatric cases, our patient shared several characteristic features, including chronic centrofacial papulopustular inflammation and prolonged diagnostic delay, as described by Douglas and Zaenglein in their pediatric case series [4]. Similar to the cases reported by Izri et al. and Guerrero-González et al., extensive investigations and empirical treatments preceded definitive diagnosis, reflecting the nonspecific clinical presentation of pediatric demodicosis [3,9]. In contrast to the crusted and more severe presentation described by Guerrero-González et al. [9], our patient exhibited a predominantly inflammatory papulopustular phenotype without systemic involvement. The pseudo-lupoid facial features observed in our case parallel the atypical morphology reported by Uzuncakmak et al., in which demodicosis mimicked cutaneous lupus in a child [5]. Ocular involvement in our patient is consistent with findings from Liang et al. and Patel et al., who highlighted recurrent blepharitis and blepharokeratoconjunctivitis as underrecognized manifestations in young patients [7,8]. Therapeutically, the favorable response to metronidazole-based regimens aligns with outcomes reported in pediatric cases treated with acaricidal therapy, including systemic or topical agents, leading to clinical resolution once the correct diagnosis was established [3,4,9].

Allergic etiologies are frequently implicated in pediatric facial dermatoses, leading to unnecessary dietary restrictions or antihistaminic regimens. However, as in our case, allergy testing often fails to yield clinically significant results. The case series by Douglas and Zaenglein emphasized this misdirection, noting that IgE panels and food avoidance strategies had little effect on lesion control [4]. The absence of urticarial or gastrointestinal symptoms further weakens the allergic hypothesis in such presentations.

Our therapeutic approach involved both cutaneous and ocular acaricidal strategies. The child was successfully treated with a compounded topical metronidazole preparation and systemic metronidazole syrup, achieving complete clinical and dermoscopic remission within three months. This outcome aligns with existing evidence supporting metronidazole’s combined antiparasitic and anti-inflammatory properties, particularly in pediatric patients where ivermectin may be contraindicated or poorly tolerated [1,2,9].

Systemic metronidazole was selected because of its recognized dual anti-inflammatory and acaricidal activity in Demodex-associated dermatoses [1,2]. Metronidazole has been shown to reduce mite density while modulating the inflammatory response triggered by Demodex antigens, supporting its role in inflammatory papulopustular presentations [1,2,11]. In pediatric demodicosis, systemic and topical metronidazole have been reported as effective and well-tolerated therapeutic options, particularly in immunocompetent children with persistent or extensive disease [3,4,9]. Although oral ivermectin is an alternative systemic agent, its use in children may require weight-based considerations and cautious selection, making metronidazole a pragmatic and well-established option in selected pediatric contexts [2,9]. The cyclic short-course regimen adopted in this case was intended to optimize efficacy while limiting cumulative exposure, as intermittent systemic therapy has been described in Demodex-related conditions with acceptable safety profiles [2,11]. Given the combined cutaneous and ocular involvement and the chronic relapsing course observed in our patient, systemic therapy was considered appropriate to achieve comprehensive mite reduction and sustained clinical remission [2,7,8,12].

For ocular management, a regimen including topical azithromycin, trehalose-based lubricants, and lid hygiene with warm compresses mirrored standard protocols described by Patel et al. and Nicholls et al. [7,12]. Although azithromycin has primarily antibacterial properties, its anti-inflammatory and anti-Demodex effects have been recognized in chronic blepharitis [12]. Maintenance therapy with adapalene and palpebral emollients helped ensure long-term stability and prevention of recurrence.

Topical therapies used for cutaneous demodicosis include permethrin, sulfur-sodium sulfacetamide, ivermectin, and benzyl benzoate [6,11], while systemic agents may include ivermectin or doxycycline depending on severity. In children, metronidazole remains a safe and effective first-line agent [9,11]. Tea tree oil, though effective in vitro, poses a risk of ocular irritation and lacks standardized pediatric safety data [13]. Reflectance confocal microscopy and ultraviolet-enhanced dermoscopy offer emerging diagnostic support, although they remain primarily research tools at this stage [1].

Altogether, this observation reinforces dermoscopy as a high-yield diagnostic tool for pediatric facial and ocular demodicosis. Its implementation can streamline diagnosis, guide treatment, and significantly reduce diagnostic delays in complex pediatric cases presenting with persistent centrofacial and eyelid inflammation.

## Conclusions

This case underscores the clinical relevance and diagnostic utility of dermoscopy in pediatric facial and ocular demodicosis. In immunocompetent children presenting with chronic centrofacial papulopustular eruptions and relapsing blepharitis, Demodex infestation should be considered, even in the absence of classic risk factors, particularly after prolonged empirical treatments. Dermoscopic identification of characteristic signs enables timely diagnosis and initiation of targeted acaricidal therapy, significantly improving outcomes. Although this report represents a single observation, it adds to the limited pediatric literature and supports further awareness and systematic dermoscopic evaluation of Demodex involvement in refractory facial and ocular inflammation. Raising awareness among pediatricians, dermatologists, and ophthalmologists is essential for recognizing this likely underdiagnosed entity.
